# Elevated neck circumference and associated factors in adolescents

**DOI:** 10.1186/s12889-015-1517-8

**Published:** 2015-03-01

**Authors:** Roberta de Lucena Ferretti, Isa de Pádua Cintra, Maria Aparecida Zanetti Passos, Gerson Luis de Moraes Ferrari, Mauro Fisberg

**Affiliations:** Adolescent Medicine Sector (Adolescent Care and Support Center) of the Pediatrics Department of the Federal University of São Paulo, São Paulo, Brazil; Department of Nursing and Nutrition of the Taubate University, São Paulo, Brazil; Center for Physical Fitness Laboratory Studies of São Caetano do Sul (CELAFISCS), São Paulo, Brazil

**Keywords:** Neck circumference, Obesity, Overweight, Adolescents

## Abstract

**Background:**

Upper body subcutaneous fat, estimated by neck circumference (NC), may present greater metabolic risk than visceral fat. The aim of this study was to determine cutoff values for NC in adolescents that identify overweight and obesity, the prevalence of elevated NC, and its association with associated factors.

**Methods:**

Cross-sectional study with adolescents from public schools in São Paulo. Anthropometric variables, blood pressure and pubertal stage were collected. Cutoff values for NC were determined by Receiver Operating Characteristic curves. A binary logistic regression was used to determine relationships between NC and associated factors.

**Results:**

Among 1668 adolescents studied, 54.92% were female. The cutoff values of NC in girls and boys that identified overweight were 31.25 and 34.25 cm, and obesity, 32.65 and 37.95 cm, respectively, and the prevalence of adolescents with high NC was 32.63% in females and 37.63% among males. NC for overweight was observed that there was an association with sex, weight, body mass index, arm, waist and thigh circumferences, pubertal stages and body fat percent (BF%). NC for obesity was found association with gender, weight, arm and thigh circumferences, and BF% (p < 0.001).

**Conclusion:**

It was concluded that there is high prevalence of elevated NC and higher risks for this outcome considering overweight and obesity, sex, weight, arm and thigh circumferences, BF%, besides being an easy and simple measure for use in clinical practice.

## Background

Overweight and obesity have a strong impact throughout the world, causing changes in metabolic parameters, which will increase the chance of occurrence of risk factors for developing cardiovascular disease, a major cause of mortality worldwide [1-5]. The high prevalence of overweight in adolescents has shown a significant increase, which certainly culminate in adults more obese and severely obese [4,6-8]. A work with 10-15 years adolescents in Sao Paulo found 23% of overweight students in public schools, and 33 % in private schools, and these two types of institution obesity was present in 8.21% and 7.83% and 9.91% of girls and 17.84% boys, respectively [9].

The fat located in the central region, visceral or subcutaneous, is strongly linked to the risk of developing diseases. Recently it has been discussed in the literature that other mechanisms or other deposits of fat and visceral fat may also contribute to the development of risk factors for cardiovascular diseases [10]. Upper body subcutaneous fat (UBSF) estimated by neck circumference (NC), may have a higher metabolic risk than abdominal visceral fat. From the anatomical viewpoint, UBSF is the only fat deposit located in a separate compartment compared to the abdominal visceral fat. Free fatty acids systemic concentration are determined primarily by the UBSF in relation to the lower subcutaneous fat, and the abdominal visceral fat, especially in obese individuals, suggesting that this fat depot may play an important role in the risk of pathogenic factors to the extent that high levels are associated with the free fat acids, insulin resistance, increased VLDL production, oxidative stress, endothelial cell dysfunction, hypertension and vascular injury [11-15].

Some studies have documented that NC may play an independent correlation of risk factors than body mass index (BMI) and waist circumference [16-19]. Preis et al. also found in their court that the NC, single marker UBSF, is a new discreet identifier pathogenic fat depot both independent as synergistically with abdominal visceral fat [20]. The importance of the classification of subjects, not just by age, but primarily by pubertal stage is due to the fact that adolescence is characterized by a life cycle where there is intense physiological changes, which will result in changes in body composition of these individuals. This study aimed to determine cutoff values for NC in adolescents that identify overweight and obesity, the prevalence of elevated NC, and its association with associated factors. The main hypothesis of this study is that within each group of elevated NC, overweight and obesity, the association between independent variables and the NC.

## Methods

Cross-sectional observational study that assessed adolescents from four public schools in the city of São Paulo. This study and the related consent were approved by Institutional Review Board of Graduate Studies and Research of the Federal of São Paulo University, Protocol No. 1959/ 09.

### Study population

Through guidance from the General Secretariat of Education of the State of São Paulo were contacted all schools suggested, based on school census of 2010, with the completion of the data collection was carried out only in schools where principals agreed and allowed the entry of researchers the educational institution. After permission directors, parents were informed and signed a consent form for adolescent participation in this study. In addition, consent form was obtained from each adolescent. The sample was non – probabilistic, convenience, which evaluated 1774 adolescents, aged 10 to 17 years old, enrolled in last year’s primary and third year of high school. Sampling procedures, anthropometric measurements, and nutritional status assessments are published elsewhere [9,21].

### Inclusion criteria

All adolescents aged 10 to 17 years old, who were not attending weight-loss program, nor taking any medication that could affect blood pressure, which were not physically disabled, and non-pregnant girls were included in the study. All students who did not address these criteria were excluded.

### Variables

#### Anthropometric data

Anthropometric measurements were taken by all previously trained professionals, using standard protocols. The collected variables were weight (kg), height (cm) by these two measures was calculated BMI by dividing weight in kilograms (kg) by height in meters (m) squared, neck (cm), waist (cm), thigh (cm), hip (cm), and arm (cm) circumferences. Standards set were used by the World Health Organization (WHO) to determine the nutritional status [22].

NC was measure by adolescent standing erect and the head positioned in the Frankfurt horizontal plane. The top edge of the tape metric was placed just below the laryngeal prominence and positioned perpendicular to the long axis of the neck at the level of the thyroid cartilage, and the circumference was measured to the value close to 0.1 cm [23]. For the assessment of body fat, were used triceps and subscapular skinfold thickness, according to standard techniques, and body fat percent (BF%) calculated according to the equations of Slaughter et al. [24] and classified by Lohman [25].

#### Blood pressure measurements

To measurement of blood pressure Measurement protocol was followed, according to the V Brazilian Guidelines on Hypertension [26]. The values of blood pressure were classified according to American Academy of Pediatrics recommendations - The fourth report on the diagnosis, evaluation, and treatment of high blood pressure in children and adolescents [27] - that recommend pressure values in percentiles (90, 95 and 99).

#### Assessment of pubertal stage

For the determination of pubertal stage was used Tanner method, using boards containing original photos [28] by the technique of self – assessment [29] of breast development for girls and genitalia for boys, being considered the prepubertal adolescents in stages 1, pubertal in stages 2-4, and postpubertal in stage 5.

#### Classification by Age group

Adolescents were also classified according to age, considering: Individuals between 10 and 12 years; between 13 and 15 years; ≥16 years. This division allowed greater homogeneity among the groups, as well as being widely used in the middle.

### Statistical analysis

To describe the profile of the sample were made frequency tables of categorical variables (gender, age) and descriptive statistics (mean, standard deviation) for continuous variables (age, height, weight, BMI, circumference, sistolic blood pressure, diastolic blood pressure, BF%). The Mann-Whitney test was used to compare variables between female and male [30].

Cutoff values of NC that identify overweight and obesity were obtained by analyzing the ROC (Receiver Operating Characteristic) curves. The best cutoff values were established in general, ie, according to sex and regardless of age or pubertal stage, and according to age group and pubertal stage [31].

Crude analysis between neck circumference and the independent variables was performed by univariate logistic regression (logistic regression: risk of belonging to the group “neck above the cutoff point”). The binary regression was used to estimate the values of chance (odds ratio-OR) and their 95% confidence intervals, with and without considering risk for neck circumference as the outcome. The final model consisted of variables with p < .20 in the adjusted analysis. Enter method was used in order to define the final model. The calculations were performed by the “software” Statistical Package for the Social Sciences (SPSS) version 20.0 and the level of significance was set at p < .05 [30].

## Results

Among 1668 adolescents evaluated, 916 (54.92%) were female, with a mean age of 14.4 ± 2.31. This study showed that 21.18% of girls and 19.95% boys were overweight and 7.56% and 10.51% were obese, respectively. The measures of dispersion mean and standard deviation of age, anthropometric measurements and blood pressure were described in Table 1.

**Table 1:** **Basic characteristics of the adolescents, mean and standard deviation (SD), according to sex**

**Parameters**	**Subjects**			
	**General**	**Female**	**Male**	***P*** Value*
**Age** (years)	14.40 ± 2.31	14.39 ± 2.32	14.42 ± 2.31	.761
**Weight** (kg)	54.25 ± 14.27	52.70 ± 11.91	56.14 ± 16.51	<.001
**Height** (cm)	159.64 ± 11.21	157.30 ± 8.45	162.48 ± 13.30	<.001
**BMI** (kg/m^2^)	21.03 ± 4.07	21.16 ± 3.89	20.88 ± 4.27	.004
**NC** (cm)	31.62 ± 4.54	30.33 ± 3.88	33.19 ± 4.80	.001
**AC** (cm)	25.06 ± 4.91	24.84 ± 4.54	25.33 ± 5.31	.384
**HC** (cm)	88.65 ± 12.04	89.81 ± 11.95	87.23 ± 12.01	<.001
**WC** (cm)	71.59 ± 11.22	70.72 ± 10.39	72.65 ± 12.08	.036
**TC** (cm)	47.34 ± 7.51	48.32 ± 7.60	46.14 ± 7.22	<.001
**SBP** (mmHg)	104.98 ± 13.46	104.02 ± 12.65	106.13 ± 14.28	<.001
**DBP** (mmHg)	67.53 ± 10.60	66.84 ± 10.49	68.35 ± 10.67	.012
**BF (%)**	27.98 ± 10.00	31.21 ± 7.97	24.04 ± 10.76	<.001

*P value refers to the Mann-Whitney test to compare variables between the sexes. BMI: body mass index; NC: neck circumference; AC: arm circumference; HC: hip circumference; WC: waist circumference; TC: tight circumference; SBP: sistolic blood pressure; DBP: diastolic blood pressure; BF%: body fat percentage.

Among the variables analyzed was observed significant difference between the sexes, with the exception of age and arm circumference. The prevalence of high sistolic blood pressure was equal to 22.19% and high diastolic blood pressure, 25.17%. It was found that 77.6% of the population has BF% above the average. Tables 2 and 3 present the results of ROC curve analysis indicating the best cutoff of adiposity, overweight and obesity, to NC, according to sex, age and pubertal stage, using as gold standard the z-score of BMI. The best values can be obtained comparing the area under the curve (AUC) for each measurement.

**Table 2:** **Cutoffs values of neck circumference (cm), according to sex, age and pubertal stage, which represent the highest sensitivity and specificity in relation to overweight**

**Sex**		**n**	AUC*	CI 95%**	**Sensib. (%)**	**Specif. (%)**	**Cuttoffs (cm)**
**Pubertal stages**							
Female	Prepubertal	34	0.937	0.856-0.999	87.5	92.3	**≥28.25**
	Pubertal	694	0.763	0.722-0.805	55.7	86.1	**≥31.35**
	Postpubertal	188	0.802	0.736-0.867	77.8	69.8	**≥31.25**
Male	Prepubertal	75	0.888	0.812-0.964	88.0	82.0	**≥29.75**
	Pubertal	655	0.693	0.649-0.737	57.3	70.4	**≥34.25**
	Postpubertal	21	0.713	0.480-0.945	100.0	43.7	**≥33.90**
**Age group (years)**							
Female	10-12	312	0.854	0.811-0.897	83.9	74.5	**≥29.35**
	13-15	283	0.799	0.737-0.860	70.9	81.7	**≥31.25**
	16-17	321	0.859	0.807-0.911	80.0	78.9	**≥31.65**
Male	10-12	250	0.865	0.821-0.910	86.7	71.2	**≥29.65**
	13-15	244	0.836	0.783-0.888	77.3	75.7	**≥33.90**
	16-17	257	0.849	0.794-0.904	81.3	80.3	**≥36.45**
**General (years)**							
Female	10-17	916	0.775	0.741-0.809	61.2	83.0	**≥31.25**
Male	10-17	751	0.690	0.649-0.730	53.3	72.8	**≥34.25**

*Area under the curve: ROC curve; **95% CI: confidence interval; Sensib: sensibility; Specif.: specificity; gold standard: z-score IMC.

**Table 3:** **Cuttoffs values of neck circumference (cm), according to sex, age and pubertal stage, which represent the highest sensitivity and specificity in relation to obesity**

**Sex**		**n**	AUC*	CI 95%**	**Sensib. (%)**	**Specif. (%)**	**Cuttoffs (cm)**
**Pubertal stage**							
Female	Prepubertal	34	0.909	0.805-0.999	100.0	87.9	**≥29.75**
	Pubertal	694	0.804	0.728-0.881	76.5	77.1	**≥31.15**
	Postpubertal	188	0.857	0.771-0.943	88.2	84.2	**≥32.65**
Male	Prepubertal	75	0.857	0.773-0.942	100.0	68.7	**≥29.75**
	Pubertal	655	0.740	0.677-0.802	40.3	94.2	**≥37.95**
	Postpubertal	21	0.375	0.161-0.589	100.0	35.0	**≥33.90**
**Age group (Years)**							
Female	10-12	312	0.831	0.755-0.908	67.7	83.6	**≥30.95**
	13-15	283	0.883	0.800-0.967	80.0	89.9	**≥32.60**
	16-17	321	0.882	0.743-0.999	92.3	83.4	**≥32.45**
Male	10-12	250	0.877	0.832-0.923	93.8	71.6	**≥30.20**
	13-15	244	0.819	0.747-0.891	88.9	61.8	**≥33.55**
	16-17	257	0.924	0.865-0.982	90.0	92.4	**≥38.45**
**General (Years)**							
Female	10-17	916	0.815	0.754-0.877	63.8	90.9	**≥32.65**
Male	10-17	751	0.712	0.654-0.770	34.2	94.5	**≥37.95**

*Area under the curve: ROC curve; **95% CI: confidence interval; Sensib: sensibility; Specif.: specificity; gold standard: z-score IMC.

**Figure 1:**
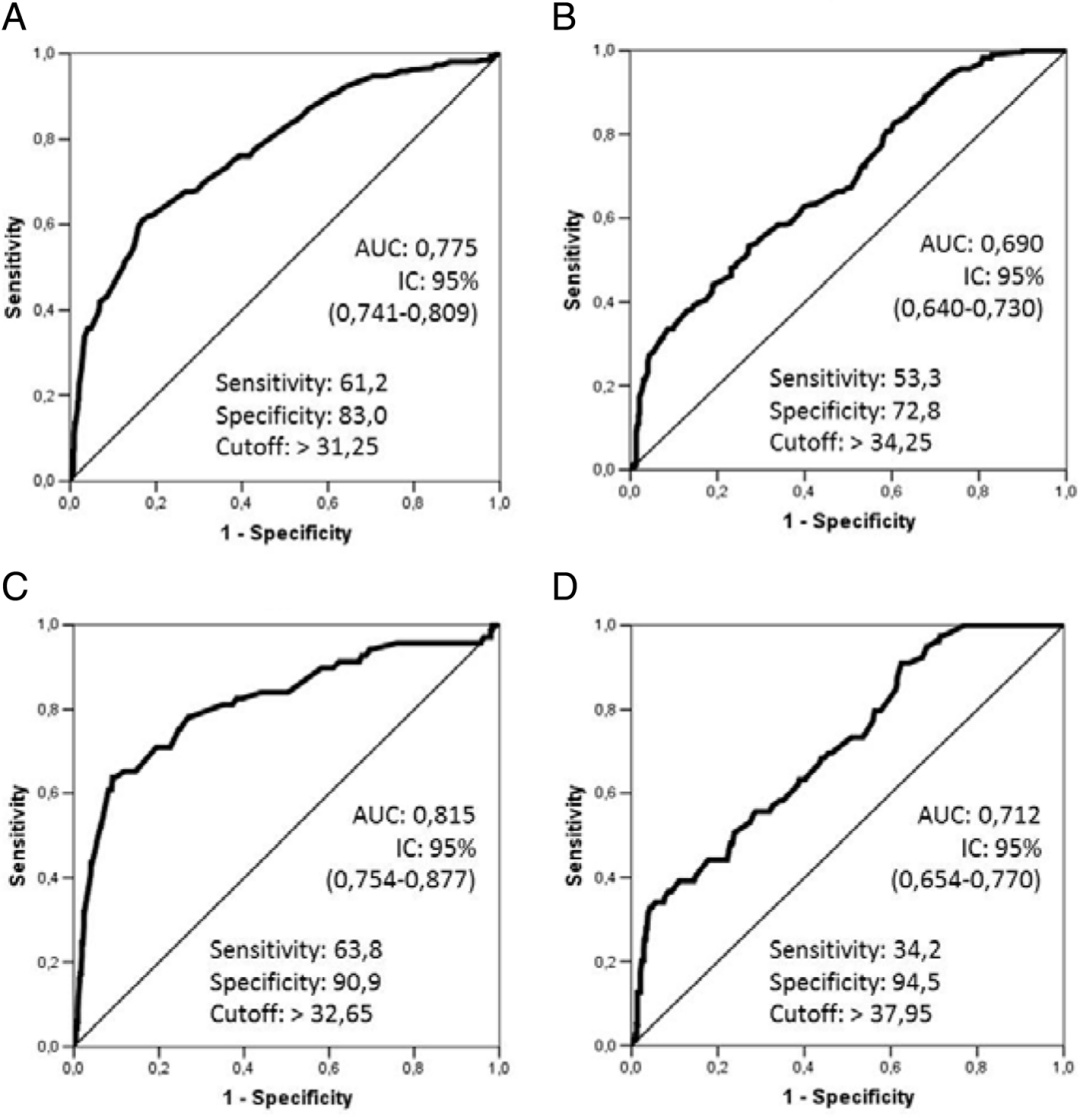
**Receiver Operator Curve of Neck Circumference.** A-Girls/Overweight; B-Boys/Overweight; C-Girls/Obesity; DBoys/Obesity”.

Considering all the adolescents studied, the cutoff values for NC that identified overweight in girls and boys were 31.25 and 34.25 cm, and obesity, 32.65 and 37.95 cm, respectively, (Figure 1) and the prevalence of individuals of both sexes, female and male, with elevated NC were 32.63 and 37.63%, respectively. For overweight, the best AUC was observed in prepubertal, both girls (AUC = 0.937; 95% CI 0.856-0.999) as boys (AUC = 0.888; 95% CI 0.812-0.964), Table 2. In relation to obesity, it was observed that the postpubertal girls showed better AUC for NC than boys in the same pubertal stage (Table 3).

Tables 4 and 5 show the univariate and adjusted logistic regression for independent variables, in overweight and obesity, respectively. After adjusting for all variables that were significantly associated with elevated NC for overweight (p <0.20), it was observed that there was an association with sex, weight, body mass index, arm, waist and thigh circumference, pubertal stage and BF% (Table 4). By analyzing the pubertal stage was observed after adjusting association in pubertal (OR = 1.59; 95% CI 1.04-2.43) and also in postpubertal (OR = 2.12, 95%CI 1.21-3.72) p = 0.002. When considering the cutoff of NC, it indicates that, in obesity, there was association of NC with adjusted sex, weight, arm and thigh circumference, and BF%.

**Table 4:** **Association between cutoff value of NC for overweight, and independent variables**

**Parameters**	**NC (Crude analysis)**		**NC (Adjusted analysis)**	
	**OR (CI 95%)**	**p**	**OR (CI 95%)**	**P**
Sex		.04*		<.001*
Female	1.00		1.00	
Male	1.24 (1.01-1.52)		0.45 (0.29-0.76)	
Group age (years)		<.001*		.191
10-12	1.00		1.00	
13-15	2.7 (1.91-4.04)		0.72 (0.25-2.12)	
>16	5.8 (4.09-8.33)		1.41 (0.46-4.32)	
Weight (kg)	1.18 (1.16-1.21)	<.001*	1.18 (1.13-1.24)	<.001*
Height (cm)	1.11 (1.09-1.12)	<.001*	0.99 (0.95-1.03)	.76
BMI classification		<.001*		.019*
Eutrophic	1.00		1.00	
Overweight	3.83 (2.97-4.94)		1.21 (0.72-2.03)	
Obesity	5.95 (4.13-8.59)		0.41(0.16-1.02)	
Arm circumference	1.55 (1.47-1.62)	<.001*	1.13 (1.04-1.22)	.002*
Hip circumference	1.09 (1.08-1.11)	<.001*	1.00 (0.99-1.05)	.761
Waist circumference	1.13 (1.11-1.15)	<.001*	1.02 (1.03-1.04)	.024*
Thigh circumference	1.21 (1.18-1.23)	<.001*	0.95 (0.92-0.99)	.037*
Pubertal stage		<.001*		
Prepubertal	1.00		1.00	.002*
Pubertal	3.42 (2.60-4.51)		1.59 (1.04-2.43)	
Postpubertal	5.65 (3.91-8.16)		2.12 (1.21-3.72)	
Sistolic blood pressure		<.001*		.22
Normotense	1.00		1.00	
Prehipertension	2.22 (1.69-2.91)		1.01 (0.66-1.56)	
Hipertension 1	2.88 (1.13-7.37)		1.87 (0.53-6.53)	
Hipertension 2	2.3 (0.86-6.19)		3.56 (0.94-13.4)	
Diastolic blood pressure		<.001*		.47
Normotense	1.00		1.00	
Prehipertension	2.39 (1.78-3.22)		1.39 (0.91-2.13)	
Hipertension 1	2.43 (1.57-3.76)		1.00 (0.52-1.94)	
Hipertension 2	2.13 (0.86-5.30)		0.83 (0.21-3.22)	
Percent body fat	1.03 (1.02-1.04)	<.001*	0.96 (0.93-0.98)	<.001*

Multivariate analysis of the association between cutoff value of NC of the adolescents, that indicates overweight, and independent variables. Logistic regression model: odds ratios (OR) and confidence intervals (95% CI). *p<.05.

**Table 5:** **Association between cutoff value of NC for obesity, and independent variables**

**Parameters**	**NC (Crude analysis)**		**NC (Adjusted analysis)**	
	**OR (CI95%)**	**p**	**OR (CI95%)**	**p**
Sex		.003*		<.001*
Male	1.00		1.00	
Female	1.61 (1.17-2.23)		8.9 (4.55-17.43)	
Group age (years)		<.001*		.180
10-12	1.00		1.00	
13-15	2.18 (1.20-3.95)		1.12 (0.51-2.44)	
>16	3.84 (2.20-6.69)		1.74 (0.75-2.44)	
Weight (kg)	1.13 (1,11-1.15)	<.001*	1.18 (1.12-1.25)	<.001*
Height (cm)	1.05 (1.03-1.07)	<.001*	0.97 (0.92-1.02)	.312
BMI classification		<.001*		
Eutrophic	1.00		1.00	.145
Overweight	6.82 (4.54-10.25)		1.70 (0.85-3.39)	
Obesity	23.51 (14.99-36.89)		3.26 (1.00-10.59)	
Arm circumference	1.38 (1.32-1.45)	<.001*	1.07 (1.01-1.13)	.008*
Hip circumference	1.02 (1.01-1.03)	.003*	0.99 (0.97-1.01)	.670
Waist circumference	1.10 (1.09-1.12)	<.001*	0.98 (0.96-1.01)	.305
Thigh circumference	1.19 (1.16-1.23)	<.001*	0.93 (0.88-0.98)	.006*
Pubertal stage		<.001*		.963
Prepubertal	1.00		1.00	
Pubertal	2.16 (1.41-3.31)		0.99 (0.53-1.84)	
Postpubertal	4.38 (2.64-7.25)		1.07 (0.50-2.29)	
Sistolic blood pressure		.010*		.134
Normotense	1.00		1.00	
Prehipertension	1.50 (1.01-2.21)		0.97 (0.54-1.73)	
Hipertension 1	3.33 (1.16-9.48)		2.57 (0.63-10.45)	
Hipertension 2	2.88 (0.91-9.07)		4.00 (1.00-16.00)	
Diastolic blood pressure		.007*		.876
Normotense	1.00		1.00	
Prehipertension	1.76 (1.17-2.64)		0.97 (0.55-1.71)	
Hipertension 1	1.86 (1.04-3.35)		0.72 (0.31-1.65)	
Hipertension 2	2.39 (0.78-7.31)		1.16 (0.25-5.42)	
Percent body fat	1.05 (1.03-1.06)	<.001*	0.97 (0.94-1.00)	.048*

Multivariate analysis of the association between cutoff value of NC of the adolescents, that indicates obesity, and independent variables. Logistic regression model: odds ratios (OR) and confidence intervals (95% CI). *p<.05.

## Discussion

It is extremely important to evaluate adolescents considering, primarily, the pubertal stage, to the extent that this is a period of intense growth and development. However, to our knowledge, this is the first study evaluating the association between elevated neck circumference in adolescents with other factors, considering all pubertal stages (prepubertal, pubertal and postpubertal).

In this study it was found that the prevalence of females and males with elevated NC were 32.63 and 37.63%, higher than the study of Guo et al., who observed elevated NC in 11.4% of girls and 23.4% of boys. However, it is important to considerer that the Guo’s study was performed with Chinese children, and this population has lower rates of overweight and obesity than the population of our study [32].

Girls and boys postpubertal, who have completed pubertal stage, showed cutoffs values of NC that identify obesity equal to 32.65/33.90 cm, respectively, values close to those established by Ben-Noun et al., in other words, NC ≥34/37 cm in adult men and women, respectively, as pointed out obesity. This comparison is due to the fact that post pubertal individuals have physiological characteristics of adulthood, and the Ben-Noun’ study was done with adults [23].

In a turkish population-based study, the AUC showed NC for prepubertal girls like this study (0.884, 95% CI 0.828-0.927 *vs* 0.937, 95% CI 0.856-0.999, respectively), as well as in pubertal girls in both studies (0.896, 95%CI 0.857-0.928 *vs* 0.763, 95% CI 0.722-0.805). Among boys, the AUC found in two studies to prepubertal were (0.899, 95% CI 0.843-0.926 *vs* 0.888, 95%CI 0.812-0.964) and the pubertal (0.877, 95% CI 0.828-0.916 *vs* 0.693 95% CI 0.649-0.737) [33]. The fact that postpubertal girls had better AUC for NC than boys in the same pubertal stage should be emphasized, however there is the small number of boys at this stage.

Cutoff values that showed higher sensitivity and specificity for NC, for overweight prepubertal and pubertal girls, were 28.25 and 31.35 cm, respectively. For obese prepubertal our cutoff was higher than Hatipoglu et al. (29.75 *vs* 28.0 cm), however for pubertal were virtually the same (31.0 *vs* 31.15 cm). As for boys, the cutoff values for overweight were 29.75 (prepubertal), and 34.25 (pubertal), and for obese the opposit occurred, in relation to girls, that is, for the prepubertal cutoff value of NC was very similar to the turkish study (29.75 vs. 29.0 cm), and, for the pubertal, our values were higher than this turkish study (37.95 vs. 32.5 cm) [33].

Importantly, in this study postpubertal girls had better AUC for NC than boys. In general, cross-sectional and longitudinal studies indicate that girls have more fat deposits than boys, especially after puberty [34-37]. However, recent and important study by Katz et al., which aimed to examine the association between NC and markers of adiposity in children, and to develop reference data on NC for the Canadian pediatric population, assessed 936 girls and 977 boys Canadians and showed that NC values for the boys were higher than the NC values for the girls, which can be explained by the fact that all individuals in overweight and obesity were excluded from the sample, which considered only the healthy-weight individuals. Among healthy-weight individuals is expected that boys have larger neck circumference than girls, especially with increasing age. However, among overweight/obese individuals that does not necessarily happen that way. So, After excluding overweight/obese, ie, into an ideal healthy population is expected that boys have larger neck circumference than girl. Also, this Canadian study did not classified participants according to pubertal stage [38].

Anthropometric measurements showing high sensitivity and specificity in predicting overweight and fat accumulation in the upper body, such as the NC, for example, are feasible. NC is simple to perform, inexpensive, has no variation in its magnitude throughout the day, it is preferable in cold weather where individuals are wearing heavy clothing, and in some cases, such as obese or morbidly obese, which feature “belly apron” or various waistlines over the abdomen [39-42].

It is important to consider that NC has good intra and inter-observer, and does not require multiple measures, for accuracy and reliability, even when compared to waist circumference. Parameters in addition to BMI have been proposed long time with the aim of better defining the body composition of an individual [43], particularly with respect to fat accumulation in the central region, which is fully associated with the development of metabolic diseases including the metabolic syndrome, characterized by central obesity, dyslipidemia, hypertension and insulin resistance, while there are doubts in terminology, for children and adolescents, there is consensus regarding cardiometabolic risk [44].

Unfortunately, the most sensitive methods to identify and complete the accumulation of body fat are expensive, difficult to be implemented in clinical practice, such as computed tomography, the DEXA (dual-energy X-ray absorptiometry), ultrasound [45]. So many authors have been engaged in developing technical protocols to better assess the distribution of body fat, especially in the pediatric age group, more cheaply and accessible to health professionals. During puberty, for example, the change in body composition is too large, depending on the stage of growth spurt, even among age-matched subjects. This makes references proposed for this assessment taking into account the pubertal stage [46]. Now there is controversy in the literature about which is the best method and technique used for the evaluation of visceral obesity in adults, stages of growth and development, such as adolescence, the difficulties are much higher [40,41].

It is widely known that the measurement of waist circumference (WC) has good ability to determine central obesity, both in adults and in children and adolescents, having already shown that in these individuals there are also good relationship with central fat [10,17,18]. In the present study the WC after being adjusted significantly correlated to overweight (p = 0.024), which did not occur when the adjustment was made in obesity (p = 0.305). In addition, WC does not have international standard classification cutoffs for classification of abdominal adiposity, much less specific to the pediatric population, and presents difficulties in measurement, that can vary significantly throughout the day, in the postprandial period in menstrual period, according to bowel function, and diverge as to the best measurement technique: the midpoint between the last intercostal arch and iliac - crest, the upper border of the iliac crest, the smallest circumference abdomen, above the umbilicus, depending on posture, respiratory phase, long after the meal [39-41].

It was observed that sex correlates significantly even when adjusted. In relation to BMI, it was found that normal individuals showed greater association with elevated NC when set this way, greater concern should be directed to these individuals, which had 2.63 greater chance to have high NC (OR: 2.63; 95%CI 1.28-5.37). Regarding circumferences, the one that presented the highest association, unilateral or adjusted, was the arm circumference (AC), in other words, the adolescent who provide high AC has a 13% more chance to present high NC (p = 0.002), and 7% chance to presenting high NC to obesity (p = 0.008), in accordance with the adjusted model. AC is important to assess subcutaneous fat and muscle mass, which may reflect a reduction in cases of malnourished individuals, or an increase in cases of obese individuals, both, respectively. In a previous study conducted with 8020 adolescents in the city of Sao Paulo, the cutoff values of the AC demonstrated for female and male high sensitivity and specificity. This shows how this measure is correlated with adiposity [9].

Although there was a significant association of NC with high blood pressure changed only in the univariate analysis, both for overweight, and for obesity, it emphasizes the importance of this issue, because it was high prevalence of this change in sistolic blood pressure (22.19%) and diastolic blood pressure (25.17%). Some studies have documented changes in blood pressure in children and adolescents (14.4%) [47-49], but less high than among adolescents in the present study. Work of Guo et al. [32], which examined whether there was an association according to nutritional status between NC and high risk of change in BP, noted that, among eutrophic participants, elevated NC was significantly associated with a greater chance for change in BP (OR = 1.637; 95% CI 1.288-2.08) in univariate analysis, and this result remained significant after adjustment for BMI and WC (OR = 1.439; 95% CI 1.118-1.853).

Study by Ferretti et al., which evaluated 917 adolescents in a specialized center in Adolescents in the city of São Paulo showed that elevated blood pressure in adolescents is highly prevalent, even among healthy-weight individuals, which can be explained by style of teenage life, characterized by high consumption of foods high in salt, sugar and fat, in addition to physical inactivity, contributing to the increase in blood pressure and various metabolic disorders, even in apparently healthy individuals [50].

To our knowledge, this is the first study to determine cutoff values for NC with Brazilian Adolescents that identify overweight and obesity and its association with associated factors. These data demonstrate the importance of NC in adolescents. However, the cross-sectional nature of this study prevents the firm causal conclusions.

Study limitations are considered, as not questioning about sleep disturbances, although the focus of this study was not sleep disturbances, recent studies have shown that sleep deprivation may be related to increased neck circumference in adults. In addition, metabolic assessment would be required, and control of internal quality of the data was not performed, intra and inter-rater reability [13,15,51].

## Conclusion

It was concluded that there is elevated prevalence of elevated NC, that shows association with other factors being higher risks for this outcome sex, weight, arm and thigh circumference and BF%, considering the cutoffs for both overweight and for obesity. NC is a great screening measure for identifying overweight in clinical practice, as well as having all the advantages of the ease of measurement, shows an association with other risk factors for chronic diseases.

## Abbreviations

AC: Arm circumference
AUC: Area under the curve
BF%: Body fat percent
BMI: Body mass index
DEXA: Dual- energy X - ray absorptiometry
HC: Hip circumference
NC: Neck circumference
OR: Odds ratio
ROC: Receiver operating characteristic
TC: Thigh circumference
UBSF: Upper body subcutaneous fat
VLDL: Very low density lipoprotein
WC: Waist circumference
WHO: World Health Organization

## References

[1] Swinburn BA, Sacks G, Hall KD, McPherson K, Finegood DT, Moodie ML (2011). The global obesity pandemic: shaped by global drivers and local environments. Lancet.

[2] Ogden CL, Carroll MD, Kit BK, Flegal KM (2012). Prevalence of obesity and trends in body mass index among US children and adolescents, 1999-2010. JAMA.

[3] Farias ES, Santos APD, Farias-Júnior JCD, Ferreira CRT, Carvalho WRGD, Gonçalves EM (2012). Excesso de peso e fatores associados em adolescentes. Rev Nutr.

[4] Rivera JA, de Cossio TG, Pedraza LS, Aburto TC, Sanchez TG, Martorell R (2014). Childhood and adolescent overweight and obesity in Latin America: a systematic review. Lancet Diabetes Endocrinol.

[5] Gee S, Chin D, Ackerson L, Woo D, Howell A (2013). Prevalence of childhood and adolescent overweight and obesity from 2003 to 2010 in an integrated health care delivery system. J Obes.

[6] The NS, Suchindran C, North KE, Popkin BM, Gordon-Larsen P (2010). Association of adolescent obesity with risk of severe obesity in adulthood. JAMA.

[7] Neovius M, Linne Y, Barkeling B, Rossner S (2004). Discrepancies between classification systems of childhood obesity. Obes Rev.

[8] Shrewsbury VA, Baur LA, Nguyen B, Steinbeck KS (2014). Transition to adult care in adolescent obesity: a systematic review and why it is a neglected topic. Int J Obes (Lond).

[9] Passos MAZ, Cintra IDP, Branco LM, Machado HDC, Fisberg M (2010). Body mass index percentiles in adolescents of the city of São Paulo, Brazil, and their comparison with international parameters. Arq Bras Endocrinol Metabol.

[10] Fox CS, Massaro JM, Hoffmann U, Pou KM, Maurovich-Horvat P, Liu CY (2007). Abdominal visceral and subcutaneous adipose tissue compartments: association with metabolic risk factors in the Framingham Heart Study. Circulation.

[11] Sjostrom CD, Hakangard AC, Lissner L, Sjostrom L (1995). Body compartment and subcutaneous adipose tissue distribution–risk factor patterns in obese subjects. Obesity Res.

[12] Nielsen S, Guo Z, Johnson CM, Hensrud DD, Jensen MD (2004). Splanchnic lipolysis in human obesity. J Clin Invest.

[13] Cizza G, Piaggi P, Lucassen EA, de Jonge L, Walter M, Mattingly MS (2013). Obstructive sleep apnea is a predictor of abnormal glucose metabolism in chronically sleep deprived obese adults. PLoS One.

[14] Horska K, Kucerova J, Suchy P, Kotolova H (2014). Metabolic syndrome - dysregulation of adipose tissue endocrine function. Ceska Slov Farm.

[15] Cizza G, de Jonge L, Piaggi P, Mattingly M, Zhao X, Lucassen E (2014). Neck circumference is a predictor of metabolic syndrome and obstructive sleep apnea in short-sleeping obese men and women. Metab Syndr Relat Disord.

[16] Laakso M, Matilainen V, Keinanen-Kiukaanniemi S (2002). Association of neck circumference with insulin resistance-related factors. Int J Obes Relat Metab Disord.

[17] Ben-Noun L, Laor A (2003). Relationship of neck circumference to cardiovascular risk factors. Obesity Res.

[18] Ben-Noun LL, Laor A (2006). Relationship between changes in neck circumference and cardiovascular risk factors. Exp Clin Cardiol.

[19] Nafiu OO, Zepeda A, Curcio C, Prasad Y (2014). Association of neck circumference and obesity status with elevated blood pressure in children. J Hum Hypertens.

[20] Preis SR, Massaro JM, Hoffmann U, D’Agostino RB, Levy D, Robins SJ (2010). Neck circumference as a novel measure of cardiometabolic risk: the Framingham Heart study. J Clin Endocrinol Metab.

[21] de Padua Cintra I, de Moraes Ferrari GL, de Sousa Vieira Soares AC, Passos MA, Fisberg M, de Souza Vitalle MS (2013). Body fat percentiles of Brazilian adolescents according to age and sexual maturation: a cross-sectional study. BMC Pediatr.

[22] de Onis M, Onyango AW, Borghi E, Siyam A, Nishida C, Siekmann J (2007). Development of a WHO growth reference for school-aged children and adolescents. Bull World Health Organ.

[23] Ben-Noun L, Sohar E, Laor A (2001). Neck circumference as a simple screening measure for identifying overweight and obese patients. Obesity Res.

[24] Slaughter MHLT, Boileau RA, Horswill CA, Stillman RJ, Van Loan MD, Bemben DA (1988). Skinfold equations for estimation of body fatness in children and youth. Hum Biol.

[25] Lohman T (1988). Anthropometric standardization reference manual.

[26] V Diretrizes Brasileiras de Hipertensão Arterial. Arquivos Brasileiros de Cardiologia Available from: http://publicacoes.cardiol.br/consenso/2006/VDiretriz-HA.pdf; 2006 [cited 2012 april 23].

[27] National High Blood Pressure Education Program Working Group on High Blood Pressure in Children and Adolescents (2004). The fourth report on the diagnosis, evaluation, and treatment of high blood pressure in children and adolescents. Pediatrics.

[28] Tanner JM (1962). Growth at Adolescence.

[29] Matsudo SMM, Matsudo VKR (1994). Self-assessment and physical assessment of sexual maturation in Brazilian boys and girls: concordance and reproducibility. Am J Hum Biol.

[30] Field A (2009). Descobrindo a estatística usando o SPSS.

[31] Jekel JF, Elmore JG, Katz DL (1996). Epidemiology, biostatistics, and preventive medicine.

[32] Guo X, Li Y, Sun G, Yang Y, Zheng L, Zhang X (2012). Prehypertension in children and adolescents: association with body weight and neck circumference. Intern Med.

[33] Hatipoglu N, Mazicioglu MM, Kurtoglu S, Kendirci M (2010). Neck circumference: an additional tool of screening overweight and obesity in childhood. Eur J Pediatr.

[34] Lee S, Kuk JL, Hannon TS, Arslanian SA (2008). Race and gender differences in the relationships between anthropometrics and abdominal fat in youth. Obesity.

[35] Loomba-Albrecht LA, Styne DM (2009). Effect of puberty on body composition. Curr Opin Endocrinol Diabetes Obes.

[36] Taylor RW, Grant AM, Williams SM, Goulding A (2010). Sex differences in regional body fat distribution from pre- to postpuberty. Obesity.

[37] Staiano AE, Katzmarzyk PT (2012). Ethnic and sex differences in body fat and visceral and subcutaneous adiposity in children and adolescents. Int J Obes (Lond).

[38] Katz SL, Vaccani JP, Clarke J, Hoey L, Colley RC, Barrowman NJ (2014). Creation of a reference dataset of neck sizes in children: standardizing a potential new tool for prediction of obesity-associated diseases?. BMC Pediatr.

[39] Aggarwal T, Bhatia RC, Singh D, Sobti PC (2008). Prevalence of obesity and overweight in affluent adolescents from Ludhiana, Punjab. Indian Pediatr.

[40] Mason C, Katzmarzyk PT (2009). Variability in waist circumference measurements according to anatomic measurement site. Obesity.

[41] Pereira PF, Serrano HMS, Carvalho GQ, Lamounier JA, Peluzio MCG, Franceschini SCC (2010). Circunferência da cintura como indicador de gordura corporal e alterações metabólicas em adolescentes: comparação entre quatro referências. Rev Assoc Med Bras.

[42] Nafiu OO, Burke C, Lee J, Voepel-Lewis T, Malviya S, Tremper KK (2010). Neck circumference as a screening measure for identifying children with high body mass index. Pediatrics.

[43] Vasques AC, Rosado L, Rosado G, Ribeiro RC, Franceschini S, Geloneze B (2010). Indicadores antropométricos de resistência à insulina. Arq Bras Cardiol.

[44] Damiani D, Kuba VM, Cominato L, Damiani D, Dichtchekenian V, Menezes Filho HCD (2011). Síndrome metabólica em crianças e adolescentes: dúvidas na terminologia, mas não nos riscos cardiometabólicos. Arq Bras Endocrinol Metab.

[45] Andreoli A, Melchiorri G, De Lorenzo A, Caruso I, Sinibaldi Salimei P, Guerrisi M (2002). Bioelectrical impedance measures in different position and vs dual-energy X-ray absorptiometry (DXA). J Sports Med Phys Fitness.

[46] Deitel M (2002). The international obesity task force and “globesity”. Obes Surg.

[47] Moore WE, Eichner JE, Cohn EM, Thompson DM, Kobza CE, Abbott KE (2009). Blood pressure screening of school children in a multiracial school district: the Healthy Kids Project. Am J Hypertens.

[48] Ejike CE, Ugwu CE, Ezeanyika LU (2010). Variations in the prevalence of point (pre)hypertension in a Nigerian school-going adolescent population living in a semi-urban and an urban area. BMC Pediatr.

[49] Rafraf M, Gargari BP, Safaiyan A (2010). Prevalence of prehypertension and hypertension among adolescent high school girls in Tabriz. Iran Food Nutr Bull.

[50] Ferretti RL, Fisberg M, Cintra IP (2012). Blood pressure in adolescents and its relationship with nutritional status. Rev Cienc Med.

[51] LaBerge RC, Vaccani JP, Gow RM, Gaboury I, Hoey L, Katz SL (2009). Inter- and intra-rater reliability of neck circumference measurements in children. Pediatr Pulmonol.

